# Health Misinformation Across Multiple Digital Ecologies: Qualitative Study of Data From Interviews With International Students

**DOI:** 10.2196/38523

**Published:** 2022-07-06

**Authors:** Rashika Bahl, Shanton Chang, Dana McKay, George Buchanan

**Affiliations:** 1 School of Computing and Information Systems University of Melbourne Parkville Australia; 2 School of Computing Technologies RMIT University Melbourne Australia

**Keywords:** international students, social media, COVID-19, misinformation, infodemic, digital ecology, health information, information seeking, web-based health information

## Abstract

**Background:**

Transient migrants such as international students have received limited support from host country governments throughout the COVID-19 pandemic. An increase in misinformation, resulting in poor health outcomes for individuals, may impact an already vulnerable group.

**Objective:**

Existing research examines the spread of misinformation. Similarly, there is extensive literature on the health information behavior of international students. However, there is a gap in the literature focusing on international students’ interaction with health misinformation. This exploratory research aims to address this gap by examining international students’ interaction with health misinformation during the COVID-19 pandemic.

**Methods:**

A total of 11 participants took part in semistructured interviews and a health misinformation-identification exercise via Zoom. The data collected were subjected to qualitative thematic analysis. Multiple rounds of coding, checked by other coders, revealed 2 themes and 6 subthemes.

**Results:**

The 2 main themes that emerged were (1) approaches to dealing with health misinformation and (2) how international students navigate across multiple digital ecologies. Results show that international students who draw on multiple digital ecologies for information reliably identify misinformation, suggesting that the use of multiple digital ecologies may have a protective effect against health misinformation.

**Conclusions:**

Findings show that international students encounter health misinformation across multiple digital ecologies, and they also compare information across multiple ecologies. This comparison may support them in identifying health misinformation. Thus, the findings of this study combat narratives of international students’ susceptibility to misinformation.

## Introduction

During the COVID-19 pandemic, there has been concern about the vulnerability of migrant groups. Transient migrants such as international students, who have temporarily migrated to another country to study [1], have received limited support from the host country governments [2]. Moreover, an increase in misinformation on social media during the COVID-19 pandemic has contributed to vaccine hesitancy among migrant groups, resulting in poor health outcomes for individuals [3]. International students, due to their transient status, may draw on multiple digital ecologies to seek out information [4,5]. Drawing on multiple digital ecologies may influence international students’ reactions to misinformation. They may experience an increased exposure to misinformation or miss out on crucial local information [6].

Existing literature covers the spread of misinformation [7-9] as well as the health information behavior of international students [10-12]. However, there is a gap in literature focusing on international students’ interaction with health misinformation. This paper aims to address this gap in literature by examining the following question: “How do international students who use multiple digital ecologies interact with COVID-19 misinformation?” To do so, this paper will first synthesize the literature on the spread of misinformation and health information behavior of international students. Next, it will describe the methodology adopted. Finally, it will present results and discuss findings in the context of the literature.

### Defining Misinformation and Social Media

“Misinformation” has a variety of synonyms in the literature, including fake news, spam, and trolling [13]. In this paper, similar to the study by Wu et al [13], we use “misinformation” in its broadest sense, including any false information regardless of the source or intent.

Social media, comprised of multiple information ecologies, refers to any internet-based or digital space where individuals can form and maintain connections as well as gather and share new information [14]. Information ecologies are systems made up of the individuals using the platforms, the technologies facilitating the platforms, and the values that drive the use of the platforms [7].

### International Students and Health Misinformation

International students may be passive users of their host country health care systems due to language barriers and possessing limited information about their new host countries [11]. COVID-19 has resulted in a transition to telehealth worldwide [15], meaning that new arrivals must learn how to navigate health care systems digitally. International students have been shown to experience disconnectedness from their host countries, and digital spaces offer a line of connection to their communities [16]. Thus, international students often transcend digital ecologies as they interact with different groups of people in different geographic locations and with different cultural values [6]. Furthermore, COVID-19 policies vary across countries, which may influence international students’ knowledge and perception of host country policies [12].

When navigating new digital ecologies, international students may turn to social media to gather health information from personal connections and have “guidance in clinical decision-making” [6,10]. They may also draw on their home country media sources for health information in their first language [10,11]. While moving through new digital ecologies for which they have limited context, international students rely on the limited information available on social media platforms to determine reliability [6]. If the international student is unaware that they are encountering misinformation, their experience will be functionally comparable to when they are encountering real information [6]; that is, if the misinformation resonates with them, they may acquire (mis)information [17,18]—information that is actually misinformation.

During the COVID-19 pandemic, there has been an increase in misinformation [19]. A study done on a sample of tweets related to COVID-19 showed that 24.8% of tweets contained misinformation, and 17.4% had unverifiable information [19]. International students who transcend digital ecologies may experience a doubling of misinformation across different digital ecologies. This can be damaging, as research shows if people view a story enough times, they are more likely to believe it, even if the story is being disproved [20]. Furthermore, research has demonstrated that context influences how misinformation is interpreted [21]. International students may encounter the same misinformation across digital ecologies. What might be framed accurately in one ecology could be framed inaccurately or in a biased manner in another. Biased information is a form of misinformation [13]. This difference in framing may influence international students’ behavior when they encounter misinformation across ecologies. Moreover, the values international students have developed in their home country’s digital ecologies may impact their misinformation encounters in the host country’s digital ecologies [6].

## Methods

### Recruitment

A total of 11 students from 3 Australian universities and 5 cultural backgrounds took part in the study.

Participants were recruited via various social and student networks. Although there was the possibility of bias, recruitment posts were public. Participants were limited to international students studying in Australia to ensure that the local context was the same for all participants. Participants were required to use 2 or more social media platforms such as Facebook, Twitter, or Weibo. However, the use of platforms and immigration status were defined by the participants. Participation was voluntary, and participants were free to withdraw at any point. Participant demographics are described in Table 1.

**Table 1:** Demographic information of research participants (N=11).

Demographics		Values, n (%)^a^
**Gender**		
	Female	6 (55)
	Male	5 (45)
**Level of study**		
	Master’s degree	7 (64)
	Bachelor’s degree	2 (18)
	PhD	2 (18)
**Discipline**		
	Information systems	5 (45)
	Engineering	2 (18)
	Commerce	2 (18)
	Medicine	1 (9)
	Public health	1 (9)
**Social media platforms used**		
	WhatsApp	11 (100)
	Facebook	11 (100)
	Instagram	9 (82)
	LinkedIn	7 (64)
	WeChat	4 (36)
	Twitter	3 (27)
	Weibo	3 (27)
	Snapchat	2 (18)
	Reddit	2 (18)
	QQ	2 (18)
	Line	2 (18)
	WeBlock	1 (9)
	Zalo	1 (9)
**Country of origin**		
	India	4 (36)
	China	3 (27)
	Indonesia	2 (18)
	France	1 (9)
	Vietnam	1 (9)

^a^Percentages have been rounded up.

### Data Collection

Data were collected in August 2021 as part of a larger study and through 3 instruments: a preinterview questionnaire, a semistructured interview, and a health misinformation-identification exercise. This research design adopts a combination of epistemological perspectives. By distinguishing the posts as information and misinformation, there is a general “truth” identified, with a post being objectively valuable because it is “true” [22]. However, especially because marginalized communities often have different experiences from those of dominant communities, truth can also be culturally constructed [23]. Interview questions were designed to elucidate how participants constructed meaning from (mis)information they came across. The objective was to understand how international students’ different backgrounds and their different cultural contexts or preexisting presumptions impacted the way they interpreted information and misinformation.

The interviews were conducted and recorded using Zoom. Each of the interviews lasted 53-87 minutes. This time duration included the health misinformation-identification exercise. Interviews were conducted until 11 were complete, when data saturation was achieved. At that point, no new information emerged from the interviews. No incentives were offered to the participants.

#### Preinterview Questionnaire

Through the preinterview questionnaire, demographic data were collected to contextualize participants’ responses during the interview. Results of this questionnaire are presented in Table 1.

#### Semistructured Interviews

Existing research has relied on interviews to gather data about users’ experience with information [9,11]. The study used a semistructured interview format, as they are the most data dense [24]. As part of a semistructured format, participants were asked preprepared interview questions and additional follow-up questions that arose. Students were prompted to consider the questions in the context of the COVID-19 pandemic. The questions are as follows:

- Where have you encountered the same information across multiple platforms before?
- What has been your experience with the way things have been reported?
- Tell me about the last time you saw a post on a social media platform that you suspected to be misleading.

The semistructured interview explored international students’ perceptions of their encounters with misleading information and the influences of inhabiting multiple digital ecologies on their consequent behaviors.

#### Health Misinformation-Identification Exercise

Previous literature has relied on misinformation-identification exercises [25,26]. This approach offered insights into how international students might actually react when encountering health misinformation.

Two social media posts from Australian sources were shown to the participants, one containing information and one containing misinformation about COVID-19. Claims were verified by searching for additional sources such as news articles and peer-reviewed journals to complement the original information sources. Reliability of both original and additional sources was cross-checked on Media Bias Fact Check [27].

Participants were not able to access additional sources to verify information. Participants were asked the following preprepared questions and occasional subsequent questions:

- Do you believe the information in this post is real or fake?
- How did you come to this conclusion?
- Would you want to share this information with other people?
- Why or why not?

### Ethics Approval

This study was approved by The University of Melbourne’s Office of Research Ethics and Integrity (2021-22022-18386-2).

### Data Analysis

Similar to previous research [28,29], qualitative thematic analysis was done on the data collected between September and October 2021. Coding of the data was done by the first author (RB) in conjunction with 2 other researchers. The first author is an international student who arrived in Australia the year the pandemic started. This author was thus already sensitized to some of the experiences reported by the participants and well placed to understand the context around the participants. However, the questions asked were open-ended and designed to get participants to share their experiences rather than reflect any assumptions the first author might have had.

The coding process was initiated by the first author by reading the transcriptions as well as the interview notes several times to become familiar with the data [30]. Subsequently, an initial round of coding was done by the first author by “asking questions about the data, making comparisons between data…and in doing so, deriving concepts to stand for those data, then developing those concepts in terms of their properties and dimensions” [24]. Similar codes were then grouped together under common themes [31]. Following rounds of refinement occurred in conversation with 2 other researchers to help mitigate bias. Analogous codes were merged to distill key ideas, and themes were finessed to allow for the best possible fit of the codes and themes with the data [32]. Transcripts of interviews and evaluation exercises were reviewed again to find examples for each code derived. The codes and examples were subsequently verified by other authors to ensure there was consensus regarding the fit of the codes with the data.

## Results

### Overview

Analysis of the data revealed 2 key themes and 6 subthemes surrounding international students’ interaction with misinformation across digital ecologies. These themes came from the concurrent analysis of data from the interviews and the exercise. Table 2 gives an overview of the data-derived themes presented in this paper. This list is not exhaustive but focuses on international students’ interactions with health misinformation across digital ecologies. Table 3 provides results of the misinformation-identification exercise.

**Table 2:** Results from interviews with international students (N=11).

Themes and subthemes			Values, n (%)^a^
**Approaches to health misinformation**			
	Ignoring health misinformation	8 (73)	
	Challenging health misinformation	5 (45)	
	Relying on trusted sources	4 (36)	
**Health misinformation across multiple digital ecologies**			
	Share health information with specific people	8 (73)	
	Evaluate health-centric posts using personal cultural values to determine reliability	7 (64)	
	Language barriers may inhibit uptake of host country’s sources of information	3 (27)	

^a^Percentages have been rounded up.

**Table 3:** Results from the health misinformation-identification exercise (N=11).

The exercise	Participant responses to each post, n (%)		
	True	False	Unsure
Post 1 (information)	6 (55)	3 (27)	2 (18)
Post 2 (misinformation)	0 (0)	10 (91)	1 (9)

#### Theme 1: Approaches to Health Misinformation

The actions international students take when they encounter health misinformation are collectively coded as “approaches to health misinformation.” This includes ignoring it, challenging it, and relying on trusted sources.

##### Ignoring Health Misinformation

The majority of international students described at least one instance of ignoring health misinformation. They described ignoring health misinformation by choosing not to open the source, not to read it, or simply scrolling past it. P1 said*,* “I think my dad…was saying something like [o]h yeah, they want to track us with COVID.…I don't really react.”

##### Challenging Health Misinformation

A little under half of the participants mentioned challenging health misinformation (5/11, 45%). Types of challenges included reporting posts, commenting on posts to share “accurate” information, or engaging the poster or sharer of misinformation in conversation. P3 said: “Most of the time, I ignore it but if it’s something that relates to his health or my family’s safety…[t]hen I will speak up about it.”

##### Relying on Trusted Sources

Over a third of international students stated relying on trusted sources (4/11, 36%). Trusted sources included friends and elected officials. P8 mentioned relying on their housemates for information about COVID-19 exposure sites, saying, “I didn't bother to check the news.…I just trusted my housemates.”

#### Theme 2: Health Misinformation Across Multiple Digital Ecologies

Health misinformation across multiple digital ecologies refers to international students’ interaction with misinformation across digital ecologies grounded by different geographic locations. Subthemes show that international students share information with specific people, evaluate health-centric posts based on personal cultural values, and experience language barriers.

##### Share Health Information With Specific People

Most participants share health information with specific people, especially if their contacts might be affected by that information. P9 mentioned sharing information from accounts they trusted during the COVID-19 outbreak and said the following:

At that time, I notice right away that COVID-19 will…go with…misinformation and then you receive information from some…prestige institution. So, it would be nice.

##### Evaluate Health-Centric Posts Using Personal Cultural Values to Determine Reliability

About two-thirds of international students evaluate health-centric posts using personal cultural values to determine reliability. They rely on information such as their biases against certain groups and their lived experiences. When evaluating the post in the exercise that contained COVID-19 misinformation, P2 stated, “It seems like something that insurance people back home would say.”

##### Language Barriers May Inhibit Uptake of Host Country Social Media

For some participants, language barriers may inhibit uptake of host country social media to gather information if they do not share a first language with locals in the host country. P4 stated, “Because…I didn’t check 7News like the news in Australia channel.…I just checked those Chinese version [*sic*], they would cover everything.” Further, P4 explained the following:

I think like even though I could speak English, I read English, but I need to translate it in my brain so when I read those news, I just want to know something so I don't want to spend too much time or too much energy on that, so it's more convenient and it's much easier.

#### Findings From the Health Misinformation-Identification Exercise

Findings from the exercise indicate that international students are generally reliable in identifying health misinformation. Of 11 students, 6 correctly identified post 1 as true, and 10 clearly identified post 2 as false. In both instances, participants drew on their experiences from their home countries to justify why they felt the post was true or false.

P10, when evaluating the first post, commented on the photo used in it, saying, “Yeah, because I saw AstraZeneca and I saw Covishield.…Covishield is the Indian version of AstraZeneca and AstraZeneca is from UK.”

P4, when evaluating the post in the exercise that contained COVID-19 information, stated, “I don't believe the US government. They are bullshit about everything.”

Generally, students who were unsure or felt that the information in post 1 was false felt so because they drew on their feelings and cultural experiences. The one student who was unsure about post 2 was concerned, as they used the same insurance referred to in the post.

Students who correctly identified the posts to be true or false drew on personal values to evaluate the content of the posts but also evaluated factors such as formatting of the posts and accounts of the posters.

## Discussion

Results of this study indicate that international students navigate health misinformation across digital ecologies, and this adds complexity to their experience with health misinformation. Overall, the findings from the study complement and extend our understanding of existing research on international students’ health information–seeking behaviors.

### International Students Traversing Health Misinformation Across Multiple Digital Ecologies

This research affirms international students’ use of multiple digital ecologies for information [4,5]. Drawing on multiple digital ecologies for information is not without challenges, and the literature highlights that language barriers may hinder international students’ health information seeking in new physical environments [10,11]. This research extends our understanding by showing that language barriers also apply to their health information seeking on social media. International students’ desire for information in a language they can easily interpret pushes them to sources like WeChat, where they can get tailored information from people similar to them [33,34], instead of relying primarily on their host country’s sources of information.

This research shows that similar to the general population [35], international students rely on a lifetime of experience and conditioning to evaluate health information they encounter. However, for international students, this conditioning happens through use of sources in the home country digital ecologies and may then be used to make decisions about health information they encounter in digital ecologies that are not native to them.

In evaluating health information, unlike other students [36], international students evaluate the source of the health information—they rely on trusted contacts. Sometimes these contacts may be personal connections, and other times they may be official sources. In the health misinformation-identification exercise, participants evaluated the social media account sharing the information by checking whether the account was verified and checking the name of the account or news outlet to determine the credibility of the post.

However, international students do not trust all contacts. Several international students reported receiving voluminous amounts of misinformation, particularly regarding COVID-19, from home-based digital sources. In response, they ignored misinformation from their home country digital sources. Although crowd correction is vital to defending against health misinformation [37], previous literature shows that people sometimes self-censor while using social media to avoid disrupting relationships [38]. Literature also shows that if the person perceives information to be biased and influential, they may challenge it to avoid repercussions [39]. By the same token, the findings show that international students challenge health misinformation if they believe there will be adverse consequences for their family’s well-being.

The health misinformation-identification exercise shows that international students can generally identify health misinformation accurately. International students’ exposure to misinformation via multiple digital ecologies affects how they evaluate and react to misinformation. The COVID-19 pandemic has not only led to an increase in misinformation on social media but has also adversely impacted international students worldwide [2]. In Australia, international students were prompted to return to their home countries by the Australian government and excluded from “all federal pandemic assistance programs, such as JobSeeker and JobKeeper” [40]. The increased adversity could have resulted in worse health outcomes for international students who draw on sources from other nations. Although the general Australian population was less likely to believe COVID-19 misinformation [41], there were reports of members of multicultural and linguistically diverse communities being susceptible to misinformation about vaccines and missing out on vital health information [42,43]. However, this study has demonstrated that students who have made the digital transition to a host country ecology compare information found in their multiple ecologies. This comparison supports them in identifying health misinformation.

Multicultural communities do not all seek out information in a similar manner, and painting their experiences with one brush is harmful. International students’ use of multiple ecologies may offer protection against the most negative effects of health misinformation. International students may be uniquely placed to identify it. Having exposure to multiple digital ecologies may do more than support connections with loved ones elsewhere—it may also ward against the biases present in a single ecology. This possibility has significant implications for our understanding of how to prevent the spread of health misinformation, not just among international students but also among other groups, and it is therefore worthy of future research.

### Implications

This paper sought to understand the nuances surrounding international students’ interaction with health misinformation. The pandemic created an unusual impetus for international students to engage with local sources of information for up-to-date news on an evolving situation.

Findings provide insight into international students’ health misinformation behaviors across digital ecologies. Further, findings reinforce that some international students face challenges in adopting host country sources of information. However, most importantly, findings combat the narrative of migrants’ susceptibility to health misinformation [3,42] by showing that for international students that draw on multiple digital ecologies, there may be a protective effect against misinformation.

In consideration of the findings, health information providers need to explore ways of collaborating with digital platforms to provide vital information in a manner that is in line with how international students access health information. In addition, health information providers might also collaborate with non–health-related organizations such as community organizations and home country organizations to provide information that is easily comprehensible by those who do not share a first language with host country locals. Moreover, health organizations and educational institutes might also incorporate digital transition training into orientation sessions to help familiarize newly arrived international students with accessing health care systems and information in the host country.
